# Strategies and implications for improving the cure rate of Novel Coronavirus Pneumonia in Wuhan

**DOI:** 10.7189/jogh.10.020302

**Published:** 2020-12

**Authors:** Yuxiao Zhang, Peiyu Cao, Jiejie Meng, Jiuyun Qiu, Qiwen Hu

**Affiliations:** 1 Global Health Institute/School of Health Sciences, Wuhan University, Wuhan, China; 2 School of Health Sciences, Wuhan University, Wuhan, China

COVID-19 has been continuously spreading around the world. As a city where COVID-19 epidemic emerged early, Wuhan has experienced all phases of the disease. The run of medical resources and rapid community spread resulted in low cure rate at the early stage of the epidemic. The cure rate significantly increased from 16% on February 21 to 92.2% on April 24 by application of stringent measures. On the one hand, these measures opened up a battlefield for “treatment of severe patients” in designated hospitals by pooling medical resources. On the other hand, they created a second battlefield for the prevention of the spread of the epidemic by setting up Makeshift(FangCang) Hospitals, isolation points and innovating the community management mode, and formed a barrier for the transformation of mild disease into severe disease. Till today, the widespread infections in other countries, still claims many lives. This article summarized the key points of the strategies in Wuhan, and provided a reference for the prevention and control of a global epidemic in the future.

## THE SITUATION IN WUHAN

As of May 4, 2020, the global average mortality rate except China was 6.97% [1]. Besides China, the cure rate of the main affected countries in the world was generally low [2,3].

**Figure 1:**
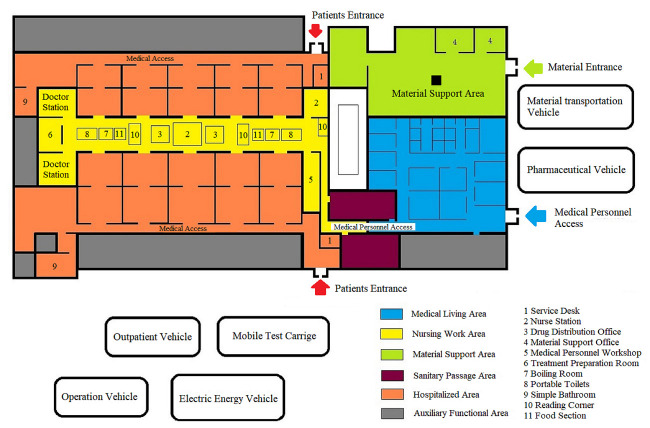
Schematic diagram of the makeshift hospital’s structure

The COVID-19 epidemic emerged early in Wuhan and the cure rate of the novel coronavirus pneumonia was low in the early stages. Implementation of a series of epidemic prevention and treatment measures caused the cure rate to improve continuously and the mortality rate to decline. The rate of severe cases showed a significant downward trend around February 26, then it became stable [4]. The number of severely and critically ill patients was all cleared at the peak of 9689 on April 24. As of April 24, the cure rate in Wuhan was 92.2%, and the cure rate of severe patients reached 88.9% (**Figure 1**) [5,6].

On average, the level of medical treatment in Wuhan remained higher than that in the most remaining cities, however, the cure rate was lower than that in Hubei province during the same period. It faced the same problems as other countries:

1. The medical run was the greatest problem. Local medical resources and protective materials were initially insufficient as the rapid development of the epidemic, and medical staff got infected in hospital continuously [7,8].
2. The resource utilization efficiency was low, the treatment couldn’t be concentrated on critically ill patients efficiently, resulting in a critical illness rate of 18%, 1.8 times that of the whole country outside the Hubei Province [9,10].
3. The spread of community outbreaks had not been contained. Despite the lock down of the city, the residents still constantly infected due to life-demand activities, resulting in new patients, further exacerbating the first two problems, which formed a vicious circle.

## MEASURES TAKEN BY WUHAN TO IMPROVE CURE RATE

To improve the cure rate,Wuhan has taken a series of measures. On the one hand, these measures opened up a battlefield for “treatment of severely ill patients” in designated hospitals by pooling medical resources. On the other hand, they created a second battlefield by setting up Makeshift (FangCang) Hospitals, isolation points and innovating the community management mode, and formed a barrier for the transformation of mild disease into severe disease. The details of these measures were listed in **Table 1**.

**Table 1:** Strategies to improve the cure rate in Wuhan

Theme	Key measures	Specific details
Cure for critical cases	Centralize resources	Integrate the national and Wuhan local medical resources, and establish a multidisciplinary team of experts.
	Improve efficiency	1. Use local medical resources to set up designated hospitals for coronavirus and transform the ward into the mode of “three partitions and two channels.”
		2. Optimize the “ICU”:
		-Set the corresponding ICU clinical standards for severe patients.
		-Provide systemic symptomatic support for critically ill patients with other illnesses.
		3. Establish various new supporting management systems.
A barrier for the transformation of mild disease into severe disease	Separate healthy people from patients with novel coronavirus pneumonia and cut off the source of infection	1. Use community service mechanism to monitor epidemic changes and help separation:
		-Register the fever and mobility of community people.
		-Carry out different separation and transportation measures for different types of people.
		-Transfer confirmed patients to receive concentrated treatment, send critical patients to designated hospitals, transport mild patients failing to enter designated hospitals to other medical institutions (including makeshift hospitals) for isolation and treatment.
		-For suspected patients and close contacts with confirmed patients, transfer severe cases to designated hospitals, mild cases to centralized isolation points.
		2.Set up a transit isolation point to strictly cut off the risk of infection and establish partitions to isolate different groups of people:
		-Isolate suspected patients and perform regular nucleic acid testing. When confirmed, patients were transferred to the hospital for treatment, and two negative nucleic acid tests allowed home isolation.
		-In the very early stage when testing resources were intense, patients with CT diagnosis and other related symptoms could go to the isolation points first.
		-Screen the close contacts as the criterion of fever person.
		-Observe the possibility of resurgence for discharged patients.
	Increase the capacity of makeshift hospitals and strengthen the comprehensive treatment of patients with mild infection	1. Treat mild patients and prevent their condition from deteriorating into severe ones.
		2. Let the treatment work energetic, keep the mild patients physically and mentally healthy.
		-Prepare reading materials, snacks, and televisions
		-Organize sport activities, like Tai Chi and a little dance.
Material support	Establish a unified medical and living material allocation system	1. Guarantee medical supplies production:
		-Coordinate the material transfer from other provinces to Wuhan
		-Encourage national manufacturing companies to resume the production for Wuhan.
		-The other cities explore the use of their local drug screening platform to track the whole supply chain of drugs and related materials for Wuhan.
		2. Guarantee supply of the living materials:
		-Collect relevant information through the materials security team and “network neighborhood” app, guarantee adequate supplies by allocate the national resource. Encourage a “group-buying” mode(people in same community purchase things online as a group, and acquired lower price as this collective buying). Motivate the e-commerce and logistics participate in this mode, people can get cheap and various materials downstairs, even the hotpot can be delivered. The risk of infection is greatly reduced by this convenient shopping mode.

## EXPAND AND OPTIMIZE TREATMENT RESOURCES FOR CRITICALLY ILL PATIENTS AND UNBLOCK CRITICAL CARE CHANNELS

### Consolidate national and local resources to respond to the epidemic

First, country deployed 344 medical teams from public hospitals, provided more than 42 000 medical workers as the support team to the Hubei Province and Wuhan. Second, besides two coronavirus-specific hospitals, there were 88 designated hospitals in Wuhan in total. These hospitals suffered from medical resources shortage and now focus on the treatment for severe patients only.

### Optimize the settings of hospitals and wards

#### ICU construction

Provided systemic symptomatic support for critically ill patients with other diseases, including hyperbaric oxygen therapy, antiviral and anti-infective treatments; on average, there were at least 10 ECMO in hospitals for critical patients.

#### Ward renovation

Designated hospitals were transformed into the mode of “three partitions and two channels”. First, two channels which couldn’t be connected were established, one for patients to enter and the other for them to leave. Second, completely isolated cleaning section, partially cleaning section, and contaminated sections were separated, and the movement of people and materials between each of these areas followed stringent disinfection specifications. Finally, the wards were transformed into a negative pressure zone.

### Improve the effect of targeted therapies

For extremely dangerous patients, the comprehensive treatment team with a multidisciplinary team of respiratory specialists, critical care medicine, cardiovascular experts implemented treatment [11]. After unified training, doctors classified the risk stratification of patients as critical, severe, moderate, and mild to focus on the most critical patients. From mid-February to March, several severe patients over the age of 90 were discharged, including eight 100-year-old patients. To improve the cure rate, hospitals set up some effective systems:

1. Condition monitoring system, by reporting patient's condition with every 4 hours and daily meeting.
2. “Case-by-case” treatment for critically ill or complicated patients.
3. Multidisciplinary participation system to ensure the professional level of the critical care operations (ECMO, hemofiltration, intubation, tracheotomy etc.).
4. Nursing system by strengthening multi-organ function support, and improving communication with patients.
5. Network consultation mechanism for the rare cases by top experts, such as academician Zhong Nanshan etc.

## STRENGTHEN PREVENTION AND SEPARATION OF PATIENTS AT SOURCE TO AVOID “DAMMED LAKE” FOR PATIENTS WITH NOVEL CORONAVIRUS

People with high risk of infection were classified into “four types of people”, namely confirmed patients, suspected patients, patients with fever who could not be ruled out of infection, and close contacts with confirmed patients.

### Enhance community strength and entire society participation

China, including Wuhan, has community-based self-governing (composed of government staff and community employee) community organizations which are primarily responsible for collecting basic information, serving special vulnerable groups, patrolling the public order etc. Residents reported their own body temperature to the local government and community workers in charge. The Headquarter opened up a 24-hour mayor hotline, app of the State Council epidemic direct, to monitor the situation. Once the government received the information asking for help, it would contact the community workers to transfer and treat them.

### Set up a transit isolation point to strictly cut off the risk of infection

As the high infection risk of the coronavirus, it was not appropriate to isolate four types of people at home. The Headquarter requisitioned around 500 unused hotels, guesthouses, and some vacant college dormitories as isolation points to avoid home infections.

### Increase the capacity of Makeshift (FangCang) hospitals and strengthen the comprehensive treatment of patients with mild infection

To deal with the shortage of medical resources, 14 makeshift hospitals were set up in 35 days, and have treated more than 12 000 patients with mild infection. Depending on the progression of the disease, approximately 2% to 5% of patients were taken to the designated hospitals to continue the treatment. A makeshift hospital had not only the necessary medical staff but also an oxygen inhaler, ventilator (CPAP), and medication guides-based medicines, to meet a large number of patients with mild infection.

**Figure:**
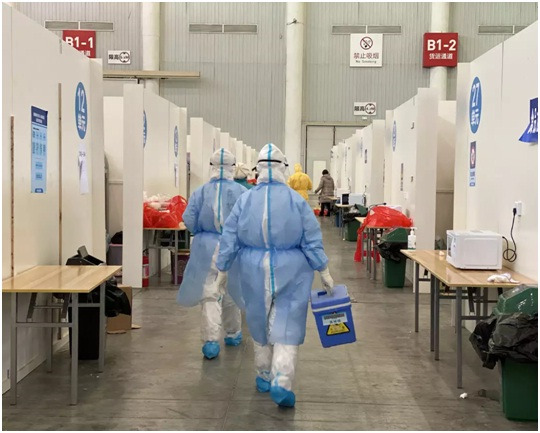
Photo: Doctors work in a makeshift hospital in Wuhan's Hanyang district (from a photographer, used with permission).

## ESTABLISH A UNIFIED MEDICAL AND LIVING MATERIAL ALLOCATION SYSTEM

### Production guarantee for medical supplies

Encouraged by national incentive policies, some manufacturing companies rebuilt and added new production lines to make the production conditions meet the standardization of medical care products. The production volume of masks ranged from 9.7 million pieces per day in early February to 116 million pieces per day in early March.

### Material support for residents

The country and the Headquarter recruited professional personnel and organizations to participate, subdivided multiple material security teams to provides residents with some cheap and free supplies.

## CONCLUSIONS

Based on the above whole strategy comb, here are a few key points for performing well to summarize.

### Subdivide various operating standards

Some practical standards are subdivided for patients’ treatment and separation, for instance:

1. Standards for admission: Patients with mild symptoms of coronavirus, between 18 and 65 years old, without basic diseases such as respiratory system, cardio-cerebrovascular system and mental illness, would be sent to makeshift hospitals. In reference to *the Diagnosis and Treatment Protocol for COVID-19* and the clinical features of patients, severe patients were sent to the designated hospitals.
2. Standards for discharge: After discharging, the patients especially the severe ones in designated hospitals would go to the isolation points for quarantine and observation to ensure the true cure.
3. Standards for setting up new hospitals: 14 Fangcang hospitals were set in 13 districts based on community population density, geographic location and therapeutic effect to ensure the distance between isolation point, makeshift hospital and designated hospital within 20-minute drive.

### Innovate the community management mode

The community not only provided living services for residents, but also served as an important barrier for front line epidemic prevention. These works have assured the life quality of residents and the normalcy of society during the epidemic, which has allowed for a stable environment in the fight against the epidemic.

### Life service management task

To meet the needs of the residents and reduce the risk of exposure, the community has taken the following measures:

1. Organized some “group buying” activities, collected the information about residents' material needs online uniformly and organized delivery downstairs, or people could choose to buy products on other platform freely. Community provided information of the free goods doled by government.
2. Supported vulnerable individuals by providing free material assistance, assigning personnel to deliver materials to their homes, taking care of the old and helping the homework with unaccompanied children etc.
3. Focused on the “non-Covid-19” patients, arranged “drug purchasing service” for patients with chronic diseases, provided the car services of going to hospitals for the patients with other severe illness.

### Community-based monitoring tasks

During the epidemic, communities have played an important role as front-line. The tasks of community in Wuhan were as follows:

1. Register the information of close contacts
2. Monitor residents’ temperature, identify and assist medical staff in transporting four groups of people.
3. Disinfect and sterilize the community regularly after the transportation of confirmed cases.
4. Timely monitor and report community outbreaks.

### Economic relevant supports

To mitigate the impact of the epidemic on the nation and people, the government have taken many measures as economic supports:

1. Strengthened fiscal policy by increasing capital investment, and ensured adequate funds for epidemic prevention in various fields. These measures included reducing the tax and providing discounted loan interest rates, providing better differentiated preferential financial services for the production of antiviral drugs and regions, industries and enterprises that were severely affected by the epidemic.
2. Motivated various business models, encouraged group-buying in community, and developed lots of Internet epidemic-related software and apps, making e-commerce and health industry-related enterprises participate in fighting the epidemic and getting good profits.
3. Realized the “zero burden” for COVID-19 patients. Costs for all kinds of tests (unlimited times), treatment (including extremely complex cases with huge expense) are paid by medical insurance and government funds. Out-of-pocket expenses relevant with COVID-19 are zero. The costs of suspected patients even not finally diagnosed are also covered. All accommodation and living expenses in Fangcang and isolation points are covered by government funds.
4. Purchased some public services. Because of various and heavy works in the community, the government attracted people to become community workers.
